# The Recycling of Spent Lithium-Ion Batteries: Crucial Flotation for the Separation of Cathode and Anode Materials

**DOI:** 10.3390/molecules28104081

**Published:** 2023-05-13

**Authors:** Xuesong Ma, Peng Ge, Lisha Wang, Wei Sun, Yongjie Bu, Miaomiao Sun, Yue Yang

**Affiliations:** 1 School of Minerals Processing and Bioengineering, Central South University, Changsha 410083, China; 2 School of Resource Environment and Safety Engineering, Hunan University of Science and Technology, Xiangtan 411201, China

**Keywords:** spent lithium-ion batteries, electrode materials, froth flotation, pretreatment

## Abstract

The recycling of spent lithium-ion batteries (LIBs) has attracted great attention, mainly because of its significant impact on resource recycling and environmental protection. Currently, the processes involved in recovering valuable metals from spent LIBs have shown remarkable progress, but little attention has been paid to the effective separation of spent cathode and anode materials. Significantly, it not only can reduce the difficulty in the subsequent processing of spent cathode materials, but also contribute to the recovery of graphite. Considering the difference in their chemical properties on the surface, flotation is an effective method to separate materials, owing to its low-cost and eco-friendly characteristics. In this paper, the chemical principles of flotation separation for spent cathodes and materials from spent LIBs is summarized first. Then, the research progress in flotation separation of various spent cathode materials (LiCoO_2_, LiNi_x_Co_y_Mn_z_O_2_, and LiFePO_4_) and graphite is summarized. Given this, the work is expected to offer the significant reviews and insights about the flotation separation for high-value recycling of spent LIBs.

## 1. Introduction

Lithium-ion batteries (LIBs) are widely used in mobile phones, laptops, cameras, and other electronic devices due to their high energy density, low self-discharge, long storage life, and safe operation [1]. In recent years, with the strong promotion and use of new energy vehicles, the growth rate of LIBs is expected to increase year by year [2]. Generally, the average lifespan of LIBs is 5–9 years, and the total weight of retired LIBs reached 355,000 tons in 2019 and is expected to reach about 800,000 tons by 2025 in China [3].

LIBs mainly consist of an anode, cathode, organic electrolyte, separator, and metal casing [4]. The anode is composed of anode active material, copper foil, and an organic binder (such as polyvinylidene fluoride (PVDF), styrene-butadiene rubber (SBR), carboxymethyl cellulose sodium (CMC), etc.). The cathode is composed of a cathode active material, conductive agent (such as carbon black), aluminum foil, and an organic binder (such as PVDF). The most commonly used anode active materials is graphite [5]. The commonly used cathode active materials include lithium metal oxides and phosphates, such as LiCoO_2_ (LCO), LiMn_2_O_4_ (LMO), LiFePO_4_ (LFP), LiNi_x_Co_y_Mn_z_O_2_(NCM), and LiNi_x_Co_y_Al_z_O_2_(NCA) [6].

Spent LIBs contain a large number of valuable elements. Taking LiNi_x_Co_y_Mn_z_O_2_ as an example, its main element composition includes Ni, Co, and Mn from the cathode active material, accounting for 14.79%, 8.49%, and 5.89%, and Cu and Al from the anode and cathode current collectors, accounting for 16.58% and 22.68%, respectively [2]. In case of the materials from spent LIBs in the environment, heavy metal pollution could be caused by metals such as Ni, Co, and Mn in the anode material, copper in the cathode, strong alkaline electrolytes, and heavy metal ions [7]. Therefore, if spent LIBs are not handled properly, they can cause environmental pollution and even harm human health. The recycling of spent LIBs has both environmental and economic benefits [8]. Different anode materials in different types of LIBs contain diverse valuable metal components, among which Co, Li, and Ni and other metals have high potential value [9]. In the future, with the continued increase in demand for high-energy-density ternary batteries, the demand for raw materials such as Co and Li will become even more scarce [10]. Therefore, recycling spent LIBs, extracting valuable metals such as Ni, Co, and Li for recycling is an effective way to avoid the risks of raw material scarcity and price fluctuations, and it has significant economic benefits.

Spent LIBs recycling processes can be mainly classified into three categories: hydrometallurgical recycling, pyrometallurgical recycling, and direct recycling. Different recovery methods have corresponding characteristics (Table 1) [11]. Pyrometallurgical technology mainly uses high-temperature smelting and reduction reactions to separate metals from impurities, and obtain metals using metallurgical methods. This technology has the advantages of simple operation, short process, and large processing capacity, and is a relatively mature recycling process [12]. However, it is prone to Li loss, requires high equipment and energy consumption, cannot achieve comprehensive recovery of cathode graphite, and generates a large amount of toxic and harmful gases. In addition, it is difficult to obtain a single metal in pyrometallurgical technology, and the resulting alloy products need to be further separated by hydrometallurgical technology [13]. Hydrometallurgical technology is an efficient metal recovery technique that can obtain relatively pure metal compounds. It includes two parts: leaching of the active substance and separation of the metal in the leachate [14]. Hydrometallurgical technology mainly uses acid–base systems to ionize the target metal ions and then uses precipitation, electrolysis, extraction, ion exchange, and other technologies to separate and enrich the metal. Finally, the target metal is recovered in the form of a certain compound [15]. Hydrometallurgical technology has the advantages of low energy consumption, high product purity, high recovery rate, and the ability to recycle various metals, making it the most favored technology for achieving the separation and purification of various elements and facilitating the subsequent recycling of valuable metals [14]. However, the leaching process of the anode material can generate waste acid, and the treatment of waste acid should be fully considered in process design. In addition, hydrometallurgical technology has a complex process, high consumption of reagents, and serious secondary pollution [14].

The pyrometallurgical process is more conducive to the large-scale industrialization of lithium battery recycling compared to the hydrometallurgical process, while hydrometallurgical technology is considered to be more sustainable due to its limited waste emissions, high metal selectivity, high recovery efficiency, and high content of value-added products [16]. Although both technologies can achieve the recycling and reuse of spent LIBs, they both focus on the recovery of metals from spent LIBs, neglecting the recovery and utilization of graphite in the black mass. Additionally, separating graphite from the black mass is also beneficial for subsequent metallurgical processing. In pyrometallurgical process, high carbon content can lead to high CO_2_ emissions, which is not in line with the development path of carbon reduction and may affect characteristics such as CO_2_/CO ratio, reaction kinetics, and melting point and viscosity of the melt, thereby affecting the metal recovery rate and process efficiency. In the hydrometallurgical process, the removal of graphite minimizes the volume of raw materials, thereby reducing the consumption of water and reagents. However, the leaching process may also damage the structure of graphite, making it unsuitable for repairing and regenerating discarded anode materials. Emerging direct recycling technologies generally refer to targeting the composition and structure of the spent materials, and selectively and non-destructively solving the problem of material failure, achieving structure regeneration, and thus restoring the electrochemical activity of the material [7]. This selective and non-destructive approach provides the possibility for regenerating electroactive materials, which can be directly reused to manufacture new LIBs. At the laboratory scale, direct recycling first involves fine recovery of the spent cathode (without cross-contamination with the anode) and supplementing the deficient lithium to improve the value of the electrode [9,17]. Lithiation reactions can be performed through reduction, solid-state, or hydrothermal methods [18]. Thermal treatment can restore the morphology and structure of the degraded cathode, recovering the cathode’s performance similar or even superior to commercial cathodes [19]. However, in most pilot and plant productions, spent batteries are crushed as a whole, resulting in mixing of electrode materials, with most of the cathode and anode active materials mixed in the black mass (<100 μm) after sieving [20]. Therefore, whether it is metallurgical or direct recycling technology, separating cathode and anode active materials from the black mass is extremely critical. Due to the inherent wettability difference between graphite and metal oxides, flotation and other processing methods can be used to separate graphite from metal oxides, further directly recycling electrode materials [17].

Flotation is a selective and non-destructive material separation method based on the physical and chemical properties of material surfaces, which can maintain the inherent structure of the material [20]. More and more researchers are applying flotation to the recovery and treatment of black mass. This article first introduces the chemical principle and feasibility of flotation separation of spent cathodes and spent lithium materials [21]. Secondly, it reviews the research progress of flotation separation of various spent cathode active materials (LiCoO_2_, LiNi_x_Co_y_Mn_z_O_2_, LiFePO_4_) and graphite, summarizes the strategies for improvement of flotation separation. Finally, it summarizes the issues that need to be addressed and provides recommendations for future research.

## 2. Flotation Principle

In this section, we discuss the wettability difference of cathode and anode graphite through their structure, introduce the principle of flotation separation, and the function of flotation agents.

### 2.1. Crystal Structure of Cathode and Anode Active Materials

Graphite is the most commonly used material in the anode. Graphite crystals have a complete layered cleavage, with the cleavage plane mainly composed of covalent bonds and a low degree of unsaturation, resulting in weak surface polarity and small dipole interactions with water molecules, which makes it naturally hydrophobic [22].

For cathode materials, LiCoO_2_ has an α-NaFeO_2_-type layered structure (R-3m space group), in which oxygen atoms are arranged in a cubic closest packing, while Li and Co occupy octahedral sites [23]. LiFePO_4_ has an olivine structure, with Li and Fe occupying octahedral sites and P located at tetrahedral positions in a slightly distorted hexagonal close packing (HCP) oxygen atom arrangement. The spinel-phase LiMnO_2_ has Li at tetrahedral sites and Mn at octahedral sites in an oxygen atom cubic closest packing (CCP) [24]. LiCoO_2_ (LCO), LiNi_x_Co_y_Mn_z_O_2_ (NCM), LiNi_x_Co_y_Al_z_O_2_ (NCA), LiFePO_4_ (LFP), and LiMn_2_O_4_ (LMO) are all ion crystals, with cleavage planes mainly composed of ionic bonds and high unsaturation bond energies, resulting in strong polarity and a strong attraction to polar water molecules, thus exhibiting strong natural hydrophilicity [22].

### 2.2. Flotation Reagent

The cathode and anode active materials, due to their significant difference in wettability, can be separated from the black mass through froth flotation [25]. Froth flotation is a separation technique based on the differences in the physical and chemical properties of minerals on their surfaces [26]. It uses flotation agents and bubbles as carriers to selectively enrich the desired minerals at the solid–liquid–gas interface, separating them from the gangue minerals. In the flotation separation process of spent LIBs, flotation reagents are mainly used for selectively adsorbing on the surface of electrode materials, increasing the wettability difference between cathode and anode active materials, and achieving flotation separation [20]. Typical flotation reagents include collectors, frothers, and regulators.

Collectors are a type of reagent that can increase the hydrophobicity of mineral surfaces and are the most important type of reagent in mineral flotation [27]. Collectors interact with active sites on the mineral surface, making it hydrophobic and adhering to the surface of the bubble, which then rises to the surface. Even for naturally hydrophobic minerals such as graphite in the flotation of black mass, non-polar oil collectors should be added appropriately to improve their floatability and separation efficiency [28]. Common collectors include kerosene, diesel, xanthates, and amines.

Frothers have a hydrophilic polar group on one end and a hydrophobic nonpolar group on the other. In froth flotation, frothers mainly adsorb at the liquid–gas interface, with the nonpolar group facing the gas phase and the polar group facing the liquid phase [29]. They form an oriented arrangement at the liquid–gas interface, reducing the surface tension of water, increasing the dispersion of air in the slurry, altering the size and motion of bubbles, and forming stable and appropriately sized foam [30]. Frothers are preferably non-collectors to facilitate process control. Common frothers include pine oil, isoamyl alcohol, and methyl isobutyl carbinol (MIBC).

Regulators mainly include pH regulators, dispersants, inhibitors, flocculants, etc. In the flotation process, the pH of the slurry is of great importance. Only under the appropriate pH conditions can various minerals be effectively floated [31]. Dispersants are compounds that have both oil-loving and water-loving properties within the molecule. They can promote the even dispersion of material particles in the medium, or uniformly disperse solid particles that are difficult to dissolve in liquids, and can also prevent the settling and agglomeration of solid particles, which are necessary for the formation of stable suspensions [32]. The applications of dispersants in froth flotation mainly involve two aspects. On the one hand, dispersants can improve the floatability of minerals by preventing the attachment of slimes (fine particles) onto mineral particles. On the other hand, they can disperse fine particles in selective flocculation. The main function of depressants is to selectively destroy or weaken the adsorption of certain minerals onto collectors and enhance the hydrophilicity of specific mineral surfaces to achieve the separation between target minerals and gangue minerals in situations where the floatability of several minerals is similar [33]. Flocculants are multifunctional group molecular organic compounds that can adsorb onto the interface between mineral particles and water in multiple points and cause flocculation. The use of selective flocculation in the froth flotation of fine-grained minerals involves changing the surface properties of target mineral particles, appropriately increasing their particle size, and then causing flocculation and separation of precipitates from gangue minerals [34].

**Figure 1:**
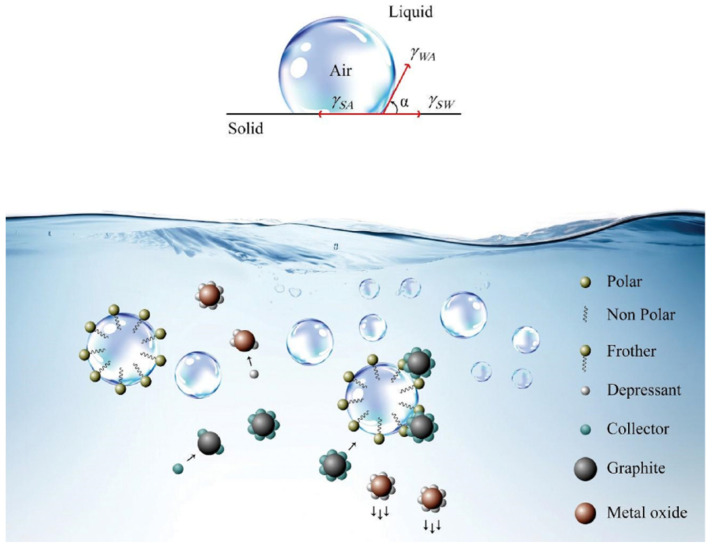
The contact angle and flotation diagram. Reprinted with permission from Ref. [35]. 2022, Elsevier.

As shown in Figure 1, flotation methods involve adding reagents and air agitation to a flotation tank containing cathode materials and anode materials (graphite). Graphite, due to its hydrophobicity, tends to distribute at the gas–liquid interface, adhere to bubbles, and float to the surface. The cathode material, due to its hydrophilicity, tends to sink to the bottom of the flotation tank [35]. Therefore, in theory, foam flotation can be used to separate them based on the difference in wettability between the cathode material and the anode material. As a non-destructive physicochemical process, foam flotation maintains the integrity of the structure and function of the cathode and anode materials, which is beneficial for reuse in new batteries after recycling treatment.

## 3. Flotation Feasibility

In order to verify the feasibility of froth flotation separation, many researchers have studied the flotation behavior of cathode and anode active materials. The grade, recovery, and yield are important indicators for evaluating the efficiency of flotation process. The grade refers to the content of valuable components in the product, which is generally determined via chemical analysis [36]. The flotation raw ore, concentrate, and tailings are represented by α, β, and θ, respectively.

Yield (γ) refers to the percentage of product mass (Q_K_) compared to the mass of the feed (Q_0_), which can be expressed as follows:

(1) $$\gamma =\frac{Q_{K}}{Q_{0}}\times 100\%$$

or

(2) $$\gamma =\frac{\alpha -\theta }{\beta -\theta }\times 100\%$$

The recovery rate is the percentage of the mass of valuable components in the product to the mass of the component in the feed, which can be expressed as follows:

(3) $$\varepsilon =\frac{\beta \times \gamma }{\alpha }\times 100\%$$

The selectivity index (SI) can be used to evaluate the flotation separation efficiency of spent LIBs, which can be expressed as follows:

(4) $$\mathrm{SI}=\sqrt{\frac{\varepsilon_{1\;I}}{\varepsilon_{2\;I}}}\times \sqrt{\frac{\varepsilon_{2\;\mathrm{II}}}{\varepsilon_{1\;\mathrm{II}}}}$$

where, SI—selectivity index; ε_1 I_—The recovery of the anode in the concentrate; ε_1 II_—The recovery of the anode in the tailings; ε_2 I_—The recovery of the cathode in the concentrate; ε_2 II_—The recovery of the cathode in the tailings. The higher the SI, the more efficient the flotation separation is.

Using a Denver 0.5 L flotation machine, Luis Verdugo et al. [21] conducted flotation experiments to investigate the flotation behavior of pure electrode materials (graphite, LCO, NCM, and NCA). For each flotation experiment, 76 g of black mass (pure graphite: pure cathode material = 47:53) was placed in a 0.5 L flotation cell and 0.5 L of water was added. To balance the hydrolysis of oxides, 0.1 M NaOH solution was added after stirring at 900 r/min for 10 min, and the slurry pH was adjusted to 12. The experiment was conducted in two groups: one without a collector and the other with 350 g/t of kerosene added as the collector. After 4 min, a frother (isoamyl alcohol, MIBC, or Aerofroth^®^ 88) was added at a concentration of 30 mg/L. Two minutes later, air was introduced at an inflation rate of 180 L/h.

**Figure 2:**
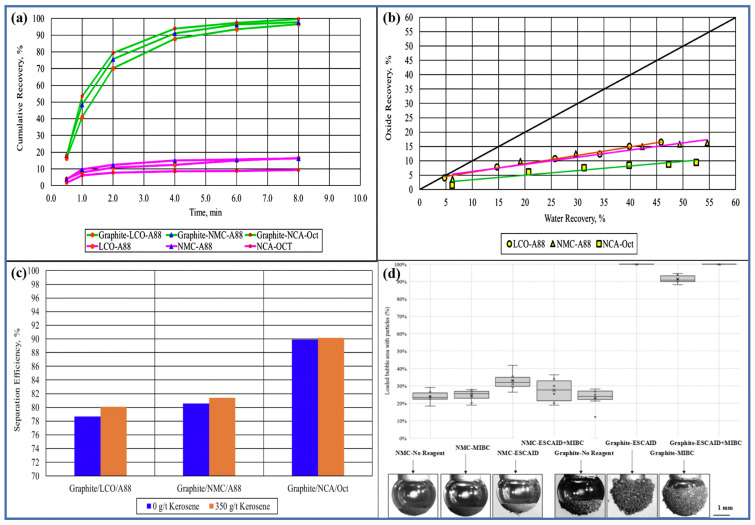
(**a**) Flotation kinetics of graphite and oxides for each frother tested, (**b**) entrainment plot for flotation tests using different frothers, (**c**) separation efficiency versus collector dosage for flotation tests with and without collector addition Reprinted with permission from Ref. [21]. 2022, Elsevier, (**d**) loaded bubble area with particles with different conditioning: no reagent, only MIBC, only ESCAID 110 and both reagents Reprinted with permission from Ref. [38]. 2021, Elsevier.

Figure 2a shows that after 8 min of the flotation process, the recovery rate of graphite is between 96.64% and 99.63%, while the recovery rate of cathode materials is between 9.47% and 16.56%. Moreover, graphite always exhibits a faster flotation rate than cathode materials. This indicates that for electrode materials that have been fully released, flotation has a good separation effect. Figure 2b shows the relationship between the recovery rate of cathode materials and the water recovery rate in the same set of experiments. This graph is called the entrainment diagram. The closer the recovery rate curve is to the 45° line, the more related the recovery rate of cathode materials is to the water recovery rate, and the higher the degree of particle entrainment [21]. These results indicate that entrainment is an important factor leading to the upward flotation of cathode materials. Figure 2c shows the effect of collector dosage on the flotation separation efficiency of different cathode materials. When no collector was used, the separation efficiency of all cathode materials exceeded 80%. When treated with 350 g/t of kerosene as a collector, the separation efficiency of all cathode materials improved slightly. Different cathode materials exhibit varying flotation separation efficiencies, which may arise from differences in crystal structure, valence bond composition leading to differences in material wettability, and differences in particle size [37].

Bubble-particle attachment experiments can serve as an indicator of particle hydrophobicity, with attachment probability depending on the physical and chemical properties of the solid particle and bubble surfaces [39]. Vanderbruggen et al. [38] conducted bubble-particle attachment experiments using pure anode active particles and natural spherical graphite or anode active particles modified with a reagent and natural spherical graphite. ESCAID 110 is a hydrocarbon fluid used as a collector at a dosage of 350 g/t during foam flotation to enhance the hydrophobicity of graphite. Methyl isobutyl carbinol (MIBC) was used as a frother at a dosage of 150 g/t. Four scenarios were designed: no reagent used, only MIBC used, only ESCAID 110 used, and both reagents used. The experimental results, shown in Figure 2d, indicate that in the absence of any reagent, some natural graphite particles were able to successfully attach to the bubbles, demonstrating their inherent hydrophobicity. Under the ESCAID 110 or ESCAID + MIBC conditions, the bubbles were completely covered by the graphite particles, indicating that ESCAID and MIBC interacted with the graphite particles, enhancing their hydrophobicity. Non-polar oil-based collectors could enhance the hydrophobicity of graphite [40]. In addition, the results showed that MIBC could also increase the area of attachment between graphite particles and bubbles. As a frother, MIBC adsorbs on the gas–liquid interface, which helps to reduce the surface tension of the liquid [36]. Then, due to its non-polar groups, it can adsorb onto both gas and solid particle surfaces, enhancing particle hydrophobicity. Miller et al. [41] and Cao et al. [42] have demonstrated that MIBC can adsorb onto coal surfaces and increase their hydrophobicity. Similar interactions between MIBC and natural graphite may also increase the hydrophobicity of graphite.

An interesting phenomenon observed in Figure 2d is that even without any reagents, some NCM can stably attach to bubbles, indicating that NCM is not completely hydrophilic. The bubble loads of NCM without and with MIBC were 23.8% ± 3.7% and 24.5% ± 3.3%, respectively. Therefore, MIBC has little influence on the wettability of NCM. However, in the presence of ESCAID 110, the bubble load of NCM was 32.8% ± 5.4%. These results support the hypothesis that ESCAID 110 interacts with NCM particles. This behavior can be explained by ESCAID 110 dispersing in the slurry as a nonpolar oil and diffusing to form an oil film upon contact with hydrophobic surfaces. As NCM is not completely hydrophilic, it may interact with nonpolar oil collectors to form an oil film that coats the particle surface, enhances particle hydrophobicity, and thus increases attachment probability [43]. Currently, most flotation separations of cathode and anode active materials from spent LIBs use nonpolar oil collectors such as kerosene and dodecane. Studies have shown that nonpolar oil collectors can interact with graphite, increasing its hydrophobicity, but their selectivity is often poor [38]. This study also demonstrates that nonpolar oil collectors can adsorb onto lithium transition metal oxides, promoting their attachment to bubbles for recovery. Therefore, studying a highly selective collector or inhibitor to improve the flotation separation efficiency of cathode and anode active materials is a promising future research direction that has been overlooked.

**Figure 3:**
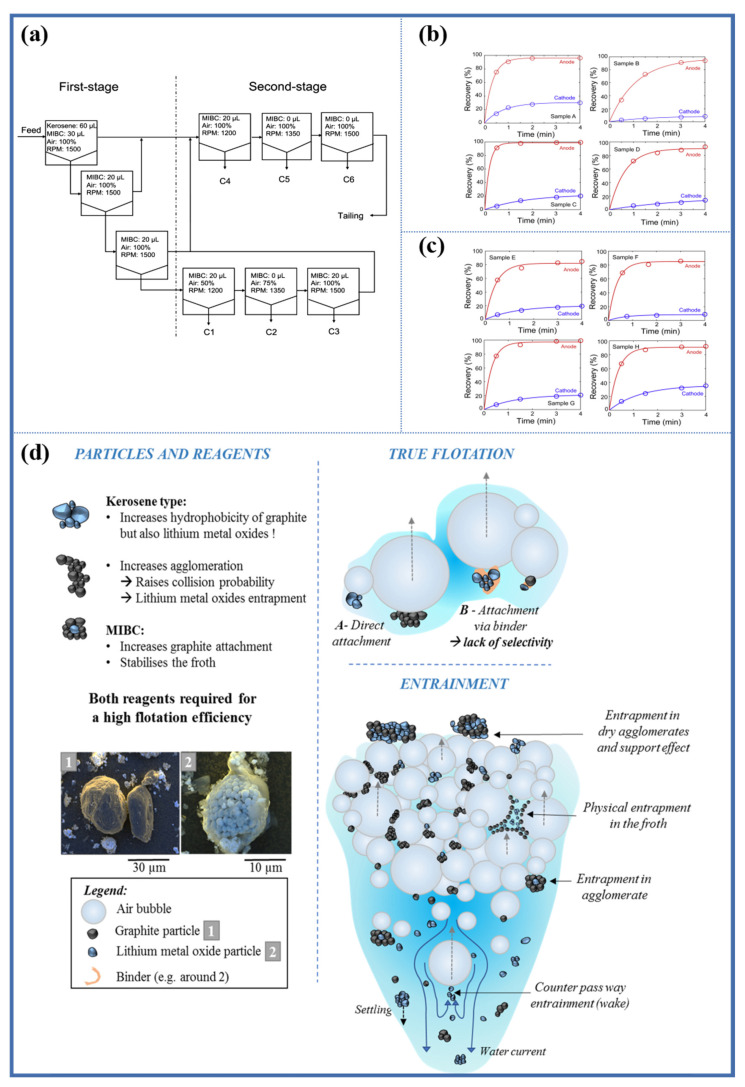
(**a**) Schematics of a modified froth flotation procedure for the separation of mixed fine materials from lithium-ion batteries, froth flotation results of individual anode and cathode electrode materials liberated from (**b**) four new lithium-ion battery samples and (**c**) four spent lithium-ion battery samples; sample A(E), B(F), C(G), D(H) are LCO, LMO, NCA, and LFP, respectively Reprinted with permission from Ref. [44]. 2018, Elsevier, (**d**) summary of simplified behavior of active particles from lithium-ion batteries during froth flotation Reprinted with permission from Ref. [38]. 2021, Elsevier.

Zhan et al. [44] designed a flotation process as shown in Figure 3a (including roughing, scavenging, and cleaning) to study the flotation behavior of different lithium-ion electrode materials at each stage. There was a significant difference in floatability between pure graphite and lithium cobalt oxide (LCO). After 4 min of flotation, the recovery rate of graphite particles exceeded 98%, while the recovery rate of lithium cobalt oxide was only 8%. The cobalt in the concentrate originated from entrainment, consistent with the results of Verdugo et al. [21]. Figure 3b shows the flotation behavior of four fresh cathode materials (LCO, LMO, NCA, and LFP) and graphite obtained from dismantled batteries. As shown in Figure 3b, all four samples of fresh cathode graphite exhibit good floatability. After the 4 min flotation process, the recovery rate of graphite was above 92.7%, while the floatability of cathode materials was poorer, with a recovery rate ranging from 8.1% to 31.0%. The differences in recovery rates among different cathode materials may be due to differences in their wettability and particle size. It is worth noting that the recovery rate of fresh cathode materials is much higher than that of pure cathode materials. For example, the recovery rate of fresh LCO particles is 27.1%, which is much higher than that of pure LCO. Such a large difference in recovery rates is not only caused by entrainment but also due to the hydrophobicity of fresh electrode materials. During the assembly process of batteries, the cathode active materials, conductive agent (such as carbon black), and binder (such as PVDF) are mixed and coated on the aluminum foil [45]. The surface of the released cathode material is covered by PVDF and carbon black, both of which are highly hydrophobic, causing an increase in the hydrophobicity of the released cathode material surface, reducing the difference in wettability between cathode and anode active particles, and increasing the recovery rate of cathode materials in the concentrate. Figure 3c shows the results of extending the foam flotation experiment to spent LIBs. It is worth noting that some electrode materials in the spent lithium-ion batteries did not adhere completely to the current collector, possibly due to the partial detachment of the binder after mechanical processing. After 4 min of flotation, the recovery rates of the anode materials ranged from 80% to 97%, while those of the cathode materials ranged from 8.8% to 35.0%. These results indicate that the hydrophobicity of the cycled anode materials has decreased. This deterioration is due to the growth of the solid electrolyte interface (SEI) on the graphite surface after cycling, which provides rich oxygen affinity sites and reduces the floatability of graphite [20].

Research has shown that the removal of PVDF can achieve the effective separation of anode and cathode active materials in black mass [44]. The presence of PVDF on the surface of electrode materials leads to a decrease in the difference in wetting properties between the anode and cathode, with average contact angles of 56° and 54°, respectively [46]. This is also the main reason why it is challenging to separate the cathode and anode materials through flotation in spent LIBs. In addition to PVDF being a challenge, there are several other factors that affect the efficiency of flotation separation of electrode materials. The charging and discharging process of a battery is actually the process of lithium ions moving back and forth between the cathode and anode. When lithium ions reach the cathode, they are embedded in the graphite layers, which may cause mechanical stress on the C-C bond and damage the graphite structure. Moreover, even with full discharge, there is still a portion of “dead lithium” in the anode. Part of this “dead lithium” comes from lithium that cannot be released from the graphite layers, and this part of the lithium will dissolve in the slurry during flotation and become impurity ions that affect flotation [47,48]. Another part comes from the SEI film produced on the graphite surface during battery cycling. The SEI film has a 20 nm oxygen-rich layer that reduces the hydrophobicity of graphite [20]. In addition, impurities in the black mass, such as copper, aluminum, iron, residual electrolyte, etc., can also affect the efficiency of flotation separation. These impurities may interact with the flotation system, thus adversely affecting flotation selectivity and flotation rate. In order to pursue higher electrochemical performance, electrode materials are often modified by surface coating, doping, structural modification, nanomaterials, etc. [49]. These methods can improve the electrochemical performance of the material, but they may also affect the flotation that is sensitive to surface properties.

In summary, there are many factors that affect the flotation separation efficiency of anode and cathode materials from black mass. Figure 3d summarizes the simplified behavior of active particles in the foam flotation process of lithium-ion batteries. Many researchers have conducted in-depth research and proposed solutions to these influencing factors. The flotation separation of different cathode materials (LCO, NCM, and LFP) and graphite will be discussed in the next section.

## 4. Strategies for Improvement of Flotation Separation

Although separating cathode and anode active materials from black mass is challenging, researchers have studied some potential solutions to achieve effective flotation separation of black mass. These solutions mainly involve pretreating the surface of black powder to increase the difference in wettability between cathode and anode active materials. Due to the different structure and properties of cathode materials, the pretreatment and flotation methods used are different.

### 4.1. LCO and Graphite Flotation Separation

**Figure 4:**
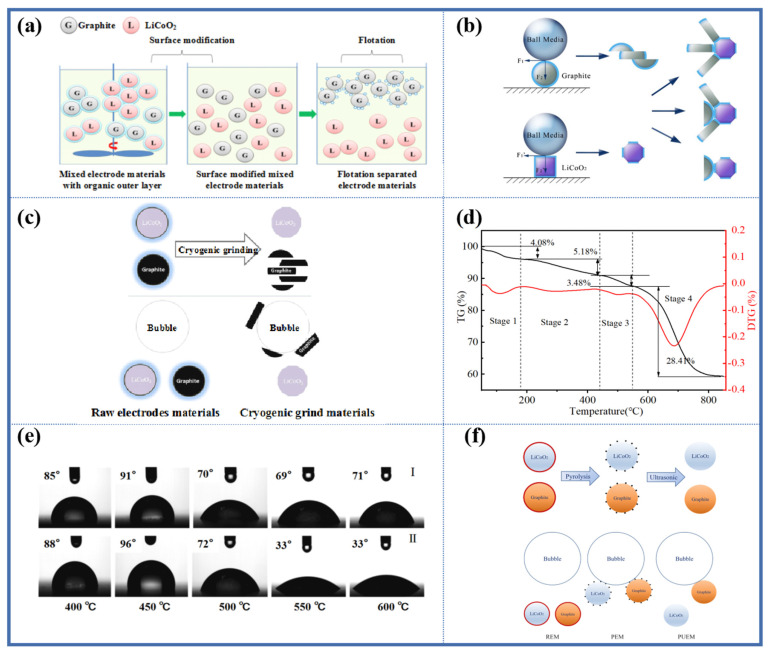
(**a**) Schematic diagram for graphite and LiCoO_2_ separation via Fenton reagent-assisted flotation Reprinted with permission from Ref. [50]. 2017, Elsevier, (**b**) dry modification mechanism based on mechanical abrasion Reprinted with permission from Ref. [52]. 2018, Elsevier, (**c**) illustration of the mechanism of flotation enhancement of LiCoO_2_ and graphite with cryogenic grinding Reprinted with permission from Ref. [53]. 2020, Elsevier, (**d**) thermogravimetric analysis of the raw spent LIB material Reprinted with permission from Ref. [54]. 2022, MDPI, (**e**) contact angles of the pyrolytic electrode materials at different pyrolysis temperatures: (I) anode material and (II) cathode material Reprinted with permission from Ref. [55]. 2021, Elsevier, (**f**) schematic diagram of LiCoO_2_ and graphite particles with different pretreatments Reprinted with permission from Ref. [56]. 2018, ACS.

A Fenton reagent-assisted flotation process was studied as shown in Figure 4a [50]. First, the Fenton reagent was used to modify the surface of black mass. In the Fenton reaction, PVDF was decomposed into small molecules, and organic matter was ultimately oxidized into CO_2_ and H_2_O. Under the optimal conditions (Fe^2+^/H_2_O_2_ ratio of 1:120 and liquid-solid ratio of 75), most of the organic coating on the electrode material surface was removed, and the content of both carbon black and PVDF decreased. LCO and graphite were exposed to their original surfaces, and the difference in wettability between the two materials increased, thus using flotation to separate LCO and graphite. The flotation performance after Fenton surface modification was unsatisfactory, with a slight improvement in the LCO grade compared to direct flotation. Further research found that although the Fenton reagent could remove the organic coating on the electrode material surface, its secondary product Fe(OH)_3_ still covered the material surface. The surface properties of the particles became similar, resulting in low flotation separation efficiency. Although adding HCl could dissolve the Fe(OH)_3_ on the material surface and eventually increase the LCO concentrate grade, H^+^ would damage the electrode material structure and cause dissolution of the cathode materials [51]. Therefore, further research is needed for the Fenton reagent-assisted flotation process.

Mechanical grinding-flotation is a physical separation method proposed for the characteristics of LCO and graphite structures [52]. As shown in Figure 4b, under the horizontal shear force generated by the grinding medium, the layered structure of graphite slides and peels off, exposing a large number of new hydrophobic surfaces. On the other hand, the organic coating on the surface of LCO is partially worn off, restoring a certain degree of hydrophilic surface. However, under the vertical rolling pressure, LCO and graphite particles will adhere to each other and become more severe with increasing grinding time. The adhesion behavior will cause LCO particles to follow graphite into the foam layer, reducing the recovery rate of LCO, but the significant wettability difference leads to an ideal LCO concentrate grade. Further research has shown that grinding can not only remove the organic coating on the surface, but also activate the lithium ions on the material surface. The activated lithium ions can destroy the C-F bond in PVDF, interact with fluoride ions to form Li-F, and increase the wettability difference between LCO and graphite. In addition, the presence of cathode particles during grinding can prevent excessive comminution of anode particles [57].

Liu et al. [53] proposed a method of low-temperature grinding-assisted flotation. At a low temperature, the organic binder exhibits a glassy state under external forces and falls off from the surfaces of LCO and graphite particles, exposing the original surface of the electrode material, as shown in Figure 4c. The grinding process has no secondary pollution and can effectively promote the efficiency of black mass flotation separation. However, this method also has some disadvantages. Firstly, the grinding process has high energy consumption, and crushing and grinding account for 30–70% of the energy input in mining [58]. Secondly, the LCO recovery rate after grinding flotation is low, and further LCO recovery is needed from the overflow product. This is mainly because the organic coating that can be removed by grinding is limited. Although low-temperature grinding can effectively improve the recovery rate of LCO, the grinding conditions are too harsh for industrial applications at present. Finally, grinding damages the structure and morphology of the electrode material, which will affect the electrochemical performance of the repaired material.

Put the black mass in the air to bake for a period of time to remove the surface organic coating. The flotation results of direct flotation, roasting modification flotation, and Fenton modification flotation were compared. Roasting modification flotation obtained the best flotation separation efficiency. The grade of LCO concentrate after roasting modification flotation is higher [51]. Proper roasting can remove organic coatings without leaving impurities on the surface of particles, fully exposing the original surface of the electrode material, and promoting the wettability difference of the cathode and anode active materials. The roasting temperature has an important influence on the flotation separation of black mass. If the temperature is too low, the organic binder and residual electrolyte on the surface of the electrode material cannot be removed. If the temperature is too high, the cathode graphite will burn. Figure 4d shows the thermogravimetric analysis (TGA) results of the black mass [54]. It can be seen that the temperature for removing the organic coating should be in the range of 450 °C to 550 °C.

Pyrolysis is a reaction process in which a substance is thermally decomposed. The pyrolysis of black mass is the process of thermally decomposing organic matter under anaerobic conditions, taking advantage of the thermal instability of organic matter. During the pyrolysis process, the organic matter undergoes chemical decomposition to produce gaseous, liquid, or solid combustibles. The pyrolysis temperature, holding time, and heating rate have an important influence on the recovery of black mass. On the one hand, if the temperature is too low, the holding time is too short, or the heating rate is too fast, the organic binder and residual electrolyte cannot be completely decomposed. On the other hand, if the temperature is too high, the pyrolysis carbon will sinter and aggregate on the surface of the electrode particles, increasing the hydrophobicity of the electrode material; and the cathode and anode materials will undergo redox reactions, destroying the electrode material structure. Zhang et al. [55,59] studied the influence of pyrolysis parameters on flotation efficiency. When the pyrolysis temperature is below 550 °C, the organic binder and pyrolysis oil cannot be completely decomposed. The hydrophobic organic binder and pyrolysis oil still adhere to the surface of electrode materials, causing an increase in the hydrophobicity of LCO particles, which makes them easy to attach to bubbles and enter the foam product. Conversely, when the pyrolysis temperature is too high, the pyrolysis carbon will sinter and accumulate on the surface of the particles, also increasing the hydrophobicity of LCO and entering the foam product. Therefore, higher or lower pyrolysis temperatures will have an adverse effect on the flotation separation efficiency. Similarly, shorter holding times and faster heating rates will also result in insufficient decomposition of the organic binder and pyrolysis oil, while longer holding times and slower heating rates will cause sintering of the pyrolysis carbon and accumulation on the surface of the electrode materials, thereby reducing flotation separation efficiency. The contact angles of the waste electrode materials at different temperatures are shown in Figure 4e. After pyrolysis flotation, the grade of LCO in the LCO concentrate was 94.72%, with a recovery rate of 83.75%. The low recovery rate of LCO may be due to the excessively fine particle size of LCO, which leads to entrainment, and the presence of pyrolysis carbon on the surface of LCO particles, which increases their hydrophobicity. Wet ball milling was performed on the black mass after 550 °C pyrolysis to remove residual pyrolysis products. After wet ball milling to remove residual pyrolysis carbon, the grade of LCO in the LCO concentrate did not change significantly, and the recovery rate increased. Therefore, wet ball milling can effectively remove pyrolysis carbon, enhance the hydrophilicity of LCO particles, and increase the recovery rate of LCO [60]. Ultrasonic cleaning can also efficiently remove residual pyrolysis products, as shown in Figure 4f [56]. The removal rates of pyrolysis products (fluorobenzene) and pyrolysis carbon using ultrasonic cleaning were 79.98% and 42.48%, respectively, efficiently cleaning the surface of electrode particles. The grade and recovery of LCO were increased using pyrolysis-ultrasonic assisted flotation.

**Figure 5:**
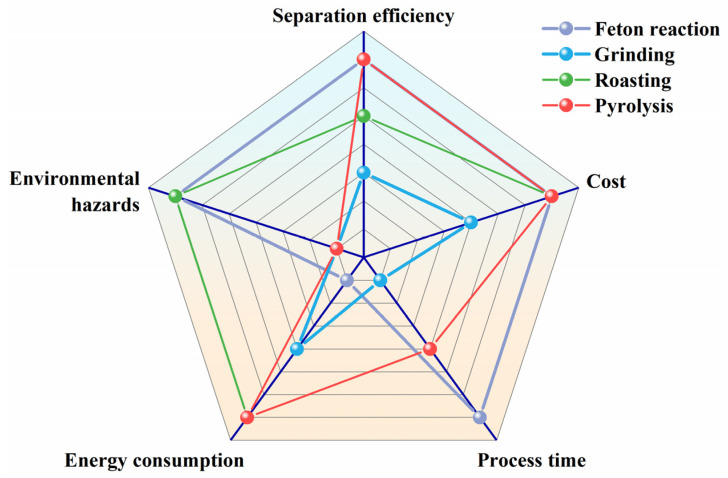
The process evaluation of different pretreatment-flotation methods.

Due to the large difference in wettability between pure LCO and graphite, flotation can easily separate them. The presence of organic coatings on the surface of spent electrode materials reduces the difference in wettability between LCO and graphite, resulting in a decrease in flotation separation efficiency [61]. Pretreatment methods such as the Fenton reaction, grinding, roasting, and pyrolysis can effectively remove organic binders and improve flotation separation efficiency [62]. The degree of removal of organic coatings directly affects the flotation separation efficiency. Figure 5 shows the process evaluation of different pretreatment-flotation methods. The Fenton reaction and ball milling are able to completely remove organic coatings but with difficulty, resulting in low flotation separation efficiency. Roasting and pyrolysis can completely remove organic coatings and achieve ideal separation effects, but roasting produce toxic gases such as HF, which is harmful to the environment, and pyrolysis requires the introduction of inert gases; moreover, the pyrolysis products accumulate on the surface of electrode materials and require further treatment, increasing costs [63]. Therefore, there is an urgent need for a clean and efficient method to remove organic coatings.

### 4.2. NCM and Graphite Flotation Separation

**Figure 6:**
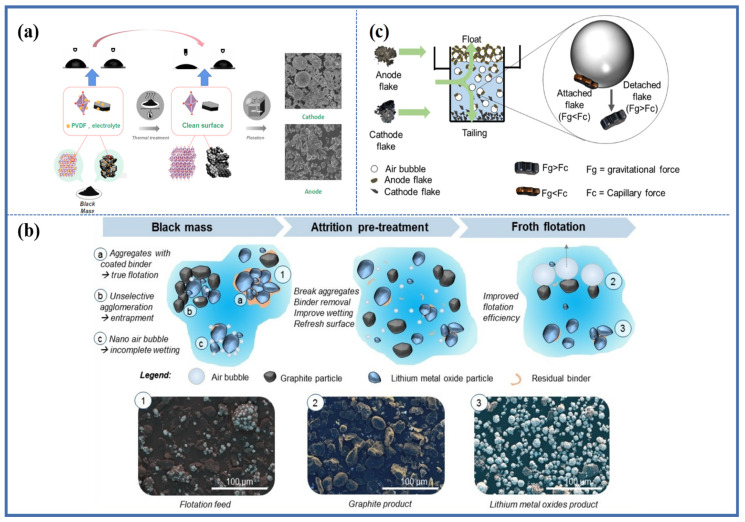
Improving separation efficiency of spent NCM and graphite by (**a**) using thermal treatment, (**b**) attrition pre-treatment Reprinted with permission from Ref. [64]. 2022, MDPI, (**c**) coarse flake particle flotation Reprinted with permission from Ref. [65]. 2023, ACS.

Ni and Mn are used to replace a portion of Co in LiCoO_2_, forming LiNi_x_Co_y_Mn_z_O_2_ (NCM) with similar structural characteristics to LiCoO_2_. Therefore, NCM has similar physical and chemical properties and wettability to LCO. Based on this, many researchers have borrowed the flotation separation method of LCO and graphite to study the flotation separation of NCM and graphite. Previous studies have shown that methods such as roasting and pyrolysis can effectively remove the organic coating on the surface of the electrode material, expose the original surface of the electrode material, increase the difference in wettability between the cathode and anode materials, and enable flotation to effectively separate the cathode and anode active materials (Figure 6a) [54,55,56,57,59,60].

After the roasting of the black mass composed of NCM and graphite particles, the organic binder and residual electrolyte on the surface of the electrode material were effectively removed, and there was no significant loss of graphite. Flotation experiments were carried out on the roasted modified black mass to investigate the effect of the collector and frother dosages on the flotation separation efficiency. After flotation, further purification operations can be carried out to obtain high-purity graphite and cathode material products, which can realize the recovery of graphite from spent LIBs [54].

Mechanical attrition pretreatment was used to improve the flotation separation efficiency of the black mass after pyrolysis. Vanderbruggen et al. [64] dispersed the pyrolyzed black powder in water with a solid content of 40%. Ultra Turrax high shear mixer was used for wear pretreatment, compared with no mechanical attrition pretreatment, the recovery rate of NCM in the bottom flow increased by 15% after flotation, while the graphite recovery rate remained unchanged. The high shear force mechanical attrition pretreatment effectively removed residual binders and improved the flotation separation efficiency by refreshing the particle surface and breaking up electrode particle aggregates, as shown in Figure 6b. This study also investigated the effect of coal oil as a collector on the flotation behavior of spent cathode and anode active materials. Coal oil increased the recovery rate of graphite and also promoted the upward flotation of NCM due to the non-selectivity of non-polar oil collectors and the incomplete hydrophobicity of NCM particles, which was confirmed in previous studies [38]. In addition, experiments were carried out without using coal oil and only using MIBC to emphasize the necessity of coal oil in spent graphite flotation. The results showed that the yield of overflow products was as low as 12%, and the graphite recovery rate was only 31%. However, under the same conditions, the recovery rate of commercial graphite was as high as 90%. These results indicate that the floatability of spent graphite is lower than that of commercial graphite, mainly ascribed to the solid electrolyte interface (SEI) film generated on the surface of anode during the charge-discharge process of LIBs [20,47]. Therefore, a collector is needed in foam flotation to enhance the floatability of spent graphite.

A new coarse-flake particle flotation technology was studied for the separation of cathode and anode active materials in black mass [65]. By taking advantage of the significant density difference between anode and cathode flake materials, effective separation and recovery of electrode flake materials could be achieved in the range of 212–850 μm, as shown in Figure 6c. It was found that the adsorption of flakes and bubbles depended on the combined action of capillary force and gravity. For larger flakes, the capillary force exceeded gravity, resulting in the electrode flakes being adsorbed onto bubbles and entering the foam layer. Conversely, when gravity exceeded capillary force, electrode flakes detached from bubbles. Therefore, maintaining an ideal feed size is crucial for flotation separation efficiency. Flotation columns were used to maintain a good feed size, this new method provides a new idea for the flotation recovery of spent LIBs from the perspective of particle structure and mechanics, without the need to remove organic binders and residual electrolytes from the surface of electrode materials.

### 4.3. LFP and Graphite Flotation Separation

**Figure 7:**
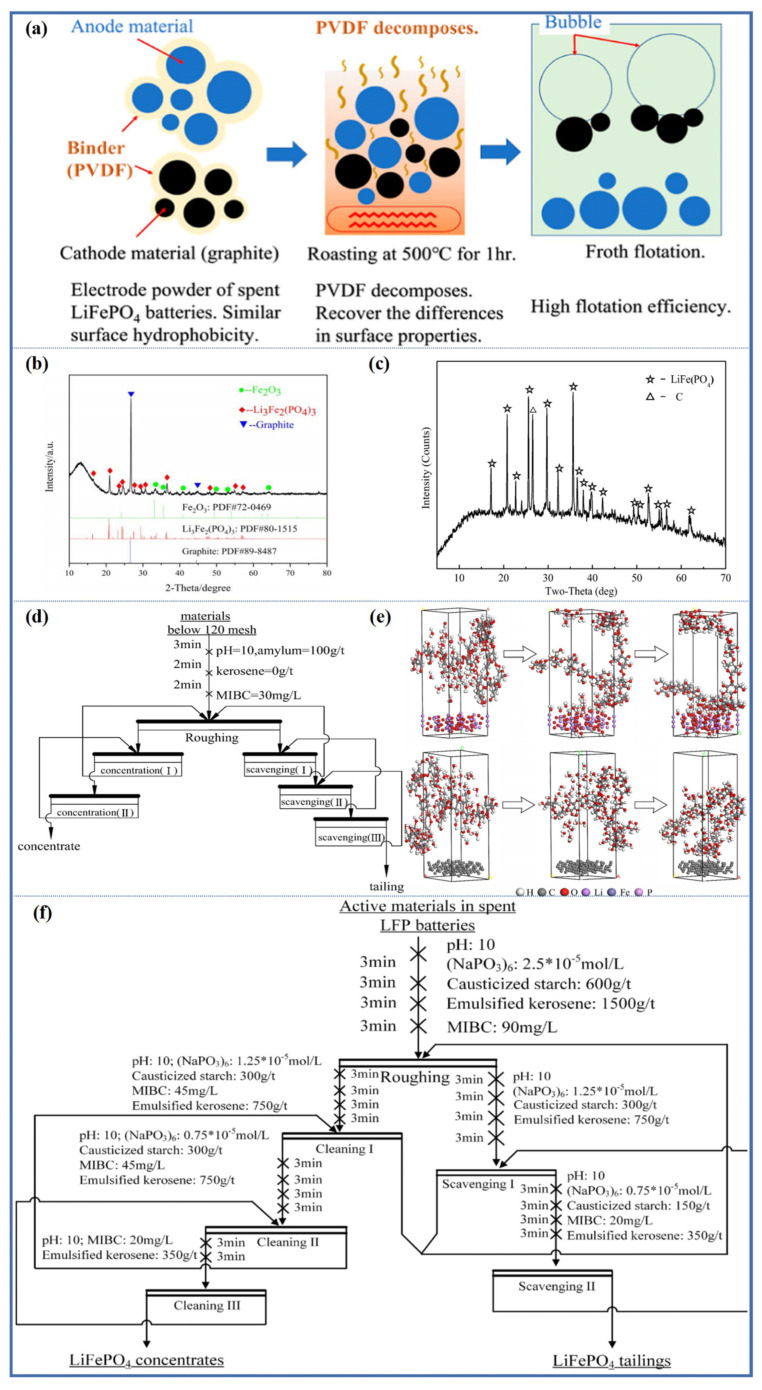
(**a**) Improving separation efficiency of spent LFP and graphite using roasting flotation, (**b**) XRD pattern of the active materials after roasting at 500 °C for 1 h Reprinted with permission from Ref. [66]. 2022, ACS, (**c**) close-circuit flowsheet of the pyrolytic flotation process, (**d**) XRD pattern of the active materials after the pyrolysis at 550 °C for 2 h Reprinted with permission from Ref. [67]. 2019, Elsevier, (**e**) closed-circuit flotation of the active materials in spent LiFePO_4_ batteries, (**f**) simulated adsorption model of soluble starch with cathode and anode active materials Reprinted with permission from Ref. [68]. 2023, Elsevier.

Thermal treatment can completely remove the organic coating on the surface of electrode material, increase the difference in wettability between LFP and graphite, and improve the flotation separation efficiency [44,54,55,56,57,59,60]. As shown in Figure 7a, the best roasting conditions for removing the organic coating on the surface of electrode material were achieved at 500 °C for 1 h. The recovery and enrichment ratios of Li after flotation were 95.87% and 1.37, respectively, while those of Fe were 95.25% and 1.36, respectively. Therefore, the roasting-flotation method is an effective process for enriching valuable metals from spent LFP batteries without wasting graphite resources. However, it is necessary to emphasize that under the best roasting conditions, the crystal structure and phase of cathode active materials have changed. The XRD test results of black mass after roasting at 500 °C for 1 h are shown in Figure 7b. As the roasting process is carried out in air, LFP is easily oxidized to generate Li_3_Fe_2_(PO_4_)_3_ and Fe_2_O_3_. Therefore, although the roasting-flotation method can effectively separate cathode and anode materials in black mass, the cathode materials cannot be directly recovered and regenerated into new batteries [66].

Pyrolysis is considered an effective method for processing spent LIBs. The pyrolysis process has the advantages of being environmentally friendly, low-cost, and simple [55,56,59,60]. Since pyrolysis is carried out in an inert atmosphere, it can effectively remove the organic coating on the surface of electrode materials, and can also prevent Fe^2+^ in spent LFP from oxidizing to Fe^3+^, which is beneficial for the repair and regeneration of cathode active materials. Zhong et al. [67] proposed a new comprehensive recycling process for spent LFP batteries with pyrolysis-assisted flotation recovery. After pyrolysis, the organic binder was removed, and the active materials could be separated from the current collector, which is beneficial for the separation of the active materials and the current collector. The XRD test results of the black mass after pyrolysis shown in Figure 7c indicate that the pyrolysis maintains the original phase of the spent cathode and anode active materials. After shearing and crushing, the cathode and anode active materials are mainly distributed in the particle range smaller than 0.25 mm, while the current collector is mainly distributed in the particle range larger than 1 mm. The flotation recovery process is shown in Figure 7d. The LFP obtained after flotation recovery was repaired and used as a new battery for electrochemical testing. The results showed that although the material had certain electrochemical properties, there was still a gap compared to commercial batteries, which may be related to the 10% impurities remaining in the recovered LFP. A large amount of LFP still exists in the overflow product and needs to be further processed.

From the above research, it can be seen that thermal treatment can effectively improve the separation efficiency of spent LFP, but it is still unable to achieve efficient separation of black mass. Therefore, more selective agents must be developed to improve the recovery of LFP. Subsequent research investigated the effect of causticized soluble starch on the flotation behavior of spent LIBs after pyrolysis [68]. The study found that causticized soluble starch can selectively adsorb on the surface of spent LFP, increase the hydrophilicity of LFP, and effectively improve the flotation separation efficiency. Theoretical calculations show that the adsorption energy between causticized soluble starch and LFP is −77,308.24 KJ/mol, while that between causticized soluble starch and graphite is −167.55 KJ/mol. Therefore, the interaction between causticized soluble starch and LFP is stronger than that between causticized soluble starch and graphite. The adsorption model of causticized soluble starch on the cathode and anode materials is shown in Figure 7e, where causticized soluble starch adsorbs slightly on the surface of graphite, while more causticized soluble starch adsorbs on the surface of LFP. After the black mass is pyrolyzed, the flotation process shown in Figure 7f is adopted, and the grade of the LFP concentrate is 84.33%, with a high recovery of 91.57%. The recovered LFP concentrate mainly consists of LFP and a small amount of graphite, which can be restored and regenerated into new anode materials. The anode material restored and regenerated is used in a new battery, exhibiting good electrochemical performance. Economic analysis shows that the cost of this process is 69.62% lower than that of traditional processes.

These recent studies indicate that with the development of new methods to improve flotation efficiency, the use of pre-treatment-flotation processes can achieve effective separation of cathode and anode active materials in black mass. Table 2 lists the advantages and disadvantages that must be considered when selecting the desired method. Generally, the selected strategy will directly depend on the envisioned future applications of the recovered materials.

## 5. Perspective and Conclusions

In the past few years, significant progress has been made in the flotation separation of anode and cathode materials of spent LIBs. In addition to the continuous maturation of the flotation technology, the enhancement technology, such as the Fenton reaction, thermal treatment, mechanical activation, and other technologies effectively removed the organic coating on the surface of electrode materials, has significantly improved the separation of spent graphite and cathode materials. Thermal treatment shows excellent application potential among them. Although flotation shows excellent application potential, there are still some problems:

(1) Fine particles are prone to be entrained. Due to the fine particle of the cathode active material, it is easy to be entrained by bubbles during the flotation process, leading to a decrease in separation efficiency. This phenomenon is particularly evident in the separation of LFP and graphite. Therefore, it is necessary to further develop efficient dispersion methods for spent anodes and cathodes to enhance flotation separation. Selective flocculation of cathode active materials seems to be a feasible method.

(2) The effect of surface modification and ion doping of electrode materials on flotation separation. Surface modification and ion doping are often used to achieve desirable electrochemical properties of electrode materials. These methods, especially surface modification, inevitably affect the wettability of the electrode material surface, which in turn affects flotation. Therefore, it is necessary to conduct in-depth research on the surface properties of electrode materials, and develop corresponding selective collectors and inhibitors to achieve efficient flotation separation.

(3) Lithium loss. As the surface of spent graphite and cathode materials often contains “death” lithium, and the electrolyte contains lithium hexafluorophosphate, these lithium resources are easy to be leached into the liquid phase by water in the form of ions during the flotation process. The lithium ion in water not only results in the loss of lithium resources, but also affect the flotation system. Therefore, how to recover lithium from beneficiation wastewater is also an important issue that needs to be addressed.

In summary, flotation separation, as an effective separation method of spent cathode material and graphite, has attracted more and more attention because of its low cost, simple operation, no chemical reaction, and material structure change. With the improvement of flotation technology, especially the recycling of mineral processing wastewater and the comprehensive recovery of metal components, flotation will become one of the most important technologies for the recovery of spent LIBs.

## Figures and Tables

**Table 1:** Comparison between the different LIB recycling processes.

	Direct Recycling	Pyrometallurgical	Hydrometallurgical
Requirements	High raw material purity	High temperatures	Acids or other precipitating agents
Recovered materials	Active materials	Raw materials	Raw materials
Advantages	Environmentally friendly High specificity; Non-destructive; Non-specific	High recycling rates; Solvent free	High recycling rates; Large variety of metals recovered
Disadvantages	Does not allow for simultaneous processing of different cathode materials	High temperatures needed; May need other processes to effectively recover materials	Complex process; Use of toxic reagents; Costly process
Efficiency evaluation	Resynthesized cell efficiency	Recovery rate	Recovery rate

**Table 2:** Advantages and disadvantages of flotation-based methods for separating cathode and graphite materials of spent LIBs.

Combined Methods	Advantages	Disadvantages	Ref.
Fenton reaction + flotation	High separation efficiency	Iron impurities	[50,51]
Grinding + flotation	No secondary pollution	Residues of the organic binders and electrolyte remaining on the electrode particles	[52,53,64]
Roasting + flotation	High efficiency and low cost	Secondary pollution caused by the combustion process	[54,66]
Pyrolysis + flotation	High separation efficiency	Need an inert atmosphere; the pyrolysis products are easy to cover the surface of electrode particles	[55,56,67,68]

## Data Availability

Not applicable.
